# Comparison of multilayer and single-layer coronary plaque models on stress/strain calculations based on optical coherence tomography images

**DOI:** 10.3389/fphys.2023.1251401

**Published:** 2023-08-07

**Authors:** Mengde Huang, Akiko Maehara, Dalin Tang, Jian Zhu, Liang Wang, Rui Lv, Yanwen Zhu, Xiaoguo Zhang, Mitsuaki Matsumura, Lijuan Chen, Genshan Ma, Gary S. Mintz

**Affiliations:** ^1^ School of Biological Science and Medical Engineering, Southeast University, Nanjing, China; ^2^ The Cardiovascular Research Foundation, Columbia University, New York, NY, United States; ^3^ Mathematical Sciences Department, Worcester Polytechnic Institute, Worcester, MA, United States; ^4^ Department of Cardiology, Zhongda Hospital, Southeast University, Nanjing, China

**Keywords:** coronary plaque, vulnerable plaque, multilayer artery model, plaque stress, imagebased plaque models

## Abstract

Mechanical stress and strain conditions are closely related to atherosclerotic plaque progression and rupture and have been under intensive investigations in recent years. It is well known that arteries have a three-layer structure: intima, media and adventitia. However, *in vivo* image-based multilayer plaque models are not available in the current literature due to lack of multilayer image segmentation data. A multilayer segmentation and repairing technique was introduced to segment coronary plaque optical coherence tomography (OCT) image to obtain its three-layer vessel structure. A total of 200 OCT slices from 20 patients (13 male; 7 female) were used to construct multilayer and single-layer 3D thin-slice models to calculate plaque stress and strain and compare model differences. Our results indicated that the average maximum plaque stress values of 20 patients from multilayer and single-layer models were 385.13 ± 110.09 kPa and 270.91 ± 95.86 kPa, respectively. The relative difference was 42.2%, with single-layer stress serving as the base value. The average mean plaque stress values from multilayer and single-layer models were 129.59 ± 32.77 kPa and 93.27 ± 18.20 kPa, respectively, with a relative difference of 38.9%. The maximum and mean plaque strain values obtained from the multilayer models were 11.6% and 19.0% higher than those from the single-layer models. Similarly, the maximum and mean cap strains showed increases of 9.6% and 12.9% over those from the single-layer models. These findings suggest that use of multilayer models could improve plaque stress and strain calculation accuracy and may have large impact on plaque progression and vulnerability investigation and potential clinical applications. Further large-scale studies are needed to validate our findings.

## 1 Introduction

Atherosclerotic cardiovascular disease is a leading cause of death worldwide, posing a significant threat to human health (Moss et al., 2019). Over the past decades, various efforts have been made to construct patient specific coronary plaque models in order to exploit efficient biomechanical outcomes and investigate the relation between plaque mechanical conditions (stress/strain) and clinical events (Yang et al., 2009; Holzapfel et al., 2014; Wang, J et al., 2020; Guo, X et al., 2021). It has been reported that plaque structural stress is a potential risk factor associated with plaque rupture. Combining plaque stress and strain with morphological factors could improve prediction accuracy of future adverse cardiovascular events (Tang et al., 2009; Stone et al., 2011; Teng et al., 2014a; Brown et al., 2016; Costopoulos et al., 2020). Additionally, endothelial shear stress is closely related to early atheroma and plaque progression, and high shear stress gradient independently predicts site of acute coronary plaque rupture and erosion (Yang et al., 2010; Samady et al., 2011; Thondapu et al., 2021).

Plaque stress/strain calculations are important for accurate predictions of plaque progression and possible clinical adverse events. It is well known that arteries have a three-layer structure: intima, media and adventitia. Intima is the innermost layer of the artery, which gradually thickens, stiffens and may develop plaques with aging and atherosclerosis. Media is the middle layer of the artery, which is a complex three-dimensional network composed of smooth muscle cells, elastin and collagen fibrils. Adventitia is the outermost layer of the artery, with low stiffness under low load, but forms a “jacket like” structure to prevent over-stretching and rupture of the artery under high pressure (Holzapfel et al., 2000). Almost all existing plaque models are single-layer models, with or without plaque component inclusions. *In vivo* image-based multilayer plaque models are not available in the current literature due to lack of multilayer image segmentation techniques and limited resolution of imaging modalities.

Optical coherence tomography (OCT) is a new imaging modality, that provides not only unprecedented resolution (5–15 μm), but also the ability to discriminate the three-layer vessel structure based on image intensity variations. A “light-dark-light” structure can be seen in OCT images with each layer showing different pixel intensities (Zahnd et al., 2017). Some studies tried several different methods to segment coronary plaque and vessel layers from OCT images, and provided the basis and potential to construct more precise multilayer coronary plaque models with useable segmented multilayer vessel image data (Olender et al., 2019; Zhang et al., 2019; Huang, M et al., 2022).

Since different arterial layers have different material and mechanical properties, incorporating layer-specific materials in multilayer models is necessary and will have a large impact on plaque stress/strain calculations. Holzapfel et al. conducted tissue stratification and axial quasi-static uniaxial tensile test on 107 samples from 9 patients with iliac artery stenosis. The experimental results showed different anisotropic tensile curves and ultimate stress of each layer (Holzapfel et al., 2004). Hoffman et al. analyzed the stiffness properties of each layer and found that adventitia was stiffer than media in both the circumferential and axial directions (Hoffman et al., 2017). Teng et al. carried out uniaxial tensile test using carotid plaques from 21 patients after endarterectomy, and found the tissue material properties are nonlinear, with media and fibrous cap harder than lipid or thrombus (Teng et al., 2014b). Holzapfel et al. studied the residual stress of each layer separately on 16 samples of 11 abdominal aortas. It was found that each layer has a different opening angle, with that of media even exceeds 180°, indicating the residual stress is layer-specific (Holzapfel et al., 2007). Many other studies have shown similar results (Gasser et al., 2006; Teng et al., 2009; Walsh et al., 2014). These studies proved the significance of developing layer-specific (or tissue-specific, location-specific) coronary models, which will lead to more accurate stress/strain calculations.

Several multilayer finite element modeling studies can be found in current literature. Teng and Brown et al. constructed two-layer 2D plaque models based on virtual histology intravascular ultrasound (Teng et al., 2014a; Brown et al., 2016). Huang, J et al. constructed three-layer 3D thin-slice plaque models based on OCT (Huang, J et al., 2021). Constant thickness was assumed for the added layers in those models. Gholipour et al. constructed an idealized three-layer fluid-structure interaction model, and found that three-layer structure of the vessel resulted in significant changes in structural stress, while shear stress remained relatively unchanged (Gholipour et al., 2018). Monir et al. segmented carotid artery with intimal thickening, incorporating stress-released geometries and the stress–strain relationships for three separate layers, to investigate the effect of layer-specific characteristics on stress in the arterial wall (Esmaeili Monir et al., 2016). These studies all indicated the importance of including three layers in the vessel/plaque models. However, they did not use patient-specific multilayer *in vivo* vessel imaging data due to lack of available segmentation techniques and segmented data.

In this paper, patient-specific multilayer thin-slice models for coronary plaques were constructed based on segmented OCT multilayer image data. Plaque stress and strain results of multilayer models were compared with single-layer models to investigate the impact of vessel three-layer structure on stress/strain calculations using three-layer models and layer-specific material properties.

## 2 Materials and methods

### 2.1 Data acquisition and processing

Twenty intravascular optical coherence tomography (OCT) coronary plaque data sets from 20 patients were used in this study. Ten existing de-identified OCT data sets with coronary heart diseases were obtained from Cardiovascular Research Foundation (CRF, New York, New York). Additional ten OCT data sets were acquired from Southeast University Affiliated Zhongda Hospital using protocol approved by Southeast University Zhongda Hospital Institutional Review Board (approval code 2019ZDKYSB046) with informed consent obtained. Patient demographic information is given in Table 1. OCT images were acquired with ILUMIEN OPTIS System and Dragonfly JP Imaging Catheter (St. Jude Medical, Westford, Massachusetts). The spatial resolution of the acquired OCT images was 4.5 μm. Slices with poor image quality were removed from this research. A total of 200 OCT slices from these patients (13 male; 7 female) were used for model construction and analysis in this study.

**TABLE 1:** Patient demographic data and clinical information. BP, blood pressure; RCA, right coronary artery; LCX, left circumflex artery; LAD, left anterior descending artery; HT, hypertension; DM, diabetes mellitus; HL, hyperlipoproteinemia.

Patient	Age	Sex	Vessel segment	BP(mmHg)	Comorbidities
P1	80	F	RCA	138/71	HT DM
P2	70	M	RCA	155/84	HT
P3	65	F	RCA	149/63	DM
P4	66	M	LCX	150/89	DM
P5	81	M	LAD	112/69	HT
P6	73	M	LCX	150/55	HT HL
P7	74	F	LAD	151/62	HT DM HL
P8	62	F	LAD	117/79	HL
P9	61	M	LCX	128/78	HT DM HL
P10	72	M	LCX	143/80	HT DM HL
P11	56	M	LAD	115/64	HT HL
P12	55	M	LAD	130/90	HT
P13	52	F	LAD	159/84	HL
P14	65	M	LAD	124/84	N/A
P15	50	F	LAD	175/92	HT HL
P16	60	M	LAD	130/84	HT HL
P17	67	F	LAD	113/60	HL
P18	67	M	RCA	136/85	HT HL
P19	60	M	LAD	141/85	HT HL
P20	32	M	LAD	101/61	N/A

### 2.2 Multilayer automatic segmentation and layer-specific material properties

**FIGURE 1:**
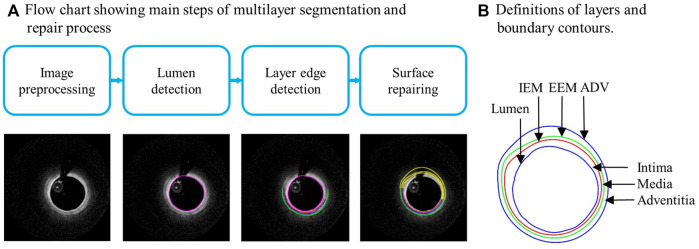
**(A)** Flow chart showing main steps of automatic multilayer segmentation and repair. **(B)** A sample slice showing definitions of lumen, three layers, and three boundary contours. IEM, Internal elastic membrane; EEM, External elastic membrane; ADV, adventitia-periadventitia interface.

**FIGURE 2:**
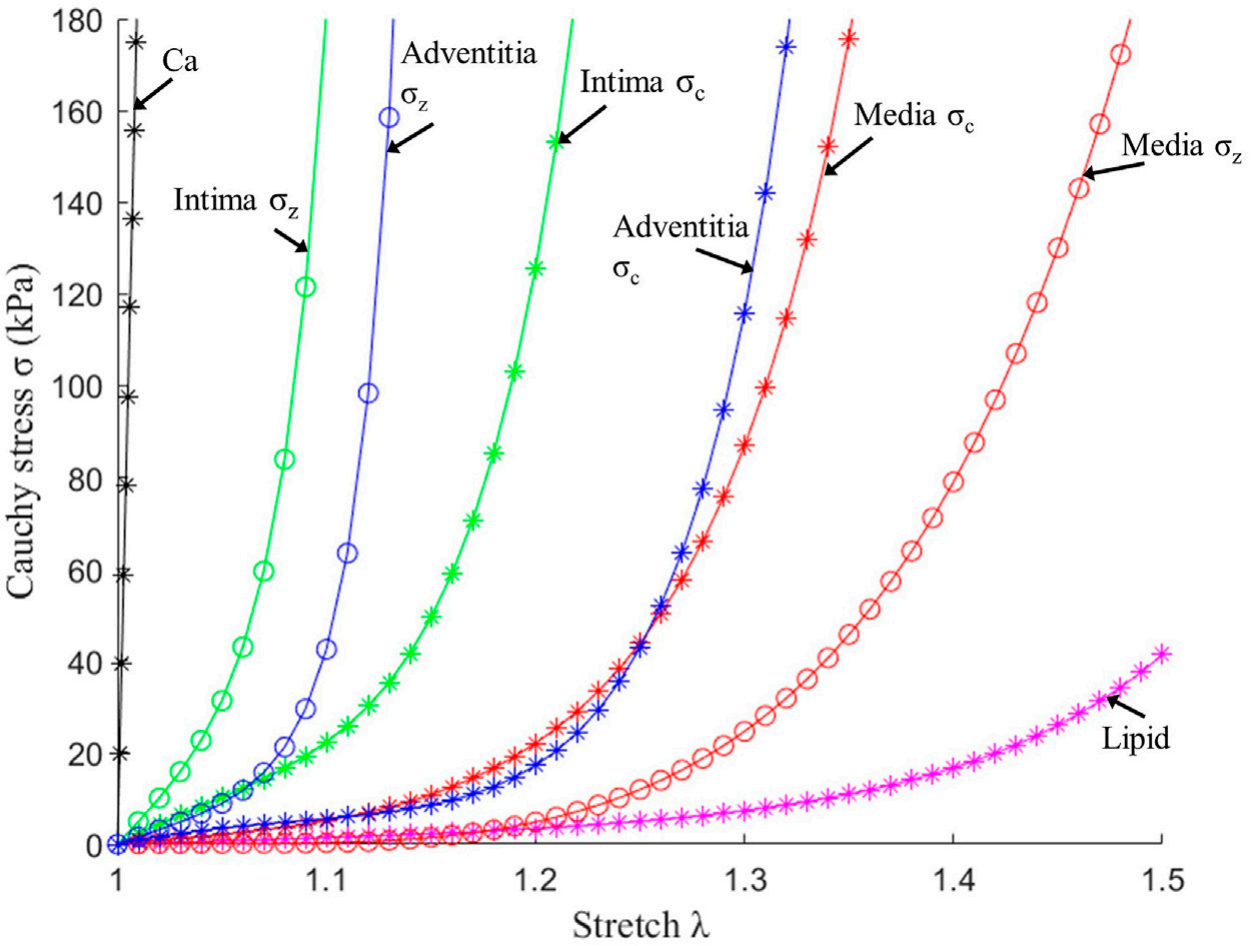
Stress–stretch curves of three layers and plaque components used in finite element modelling. σ_c_: Circumferential stress; σ_z_: Axial stress.

Multilayer automatic segmentation was performed by a method previously introduced using codes based on MATLAB (MATLAB R2021a, MathWorks, United States) (Huang, M et al., 2022). Figure 1 gives a flow chart of the multilayer automatic segmentation process and a sample slice showing the 3 layers from OCT image. The segmented multilayer (intima, media, and adventitia) image data were used for multilayer and single-layer (three layers combined) model constructions. Vessel material properties for the 3 layers were assumed to be hyperelastic, anisotropic, nearly incompressible, and homogeneous. Plaque components (lipid and calcifications) were assumed to be isotropic. Modified Mooney–Rivlin material models were used in our models with material parameter values selected to match layer-specific material curves available in the current literature (Holzapfel et al., 2005):

$$W_{iso}=c_{1}\left(I_{1}-3\right)+c_{2}\left(I_{2}-3\right)+D_{1}\left[\exp\left(D_{2}\left(I_{1}-3\right)\right)-1\right]$$ (1)

$$W_{aniso}=W_{iso}+\frac{K_{1}}{K_{2}}\left\{\exp\left[K_{2}{\left(I_{4}-1\right)}^{2}\right]-1\right\}$$ (2)

where $I_{1}=\sum \left(C_{ii}\right)$ , $I_{2}=\frac{1}{2}\left[I_{1}^{2}-C_{ij}C_{ij}\right]$ , $I_{1}$ and $I_{2}$ are the first and second invariants of right Cauchy–Green deformation tensor $C=\left[C_{ij}\right]=F^{T}F$ , $F=\left[F_{ij}\right]=\left[\partial x_{i}/\partial a_{j}\right]$ ; $\left(x_{i}\right)$ is current positionl $\left(a_{j}\right)$ is original position; $I_{4}={\lambda_{\theta }}^{2}\cos^{2}\varphi +{\lambda_{z}}^{2}\sin^{2}\varphi$ , where $\lambda_{\theta }$ , $\lambda_{z}$ are the principal stretches associated with circumferential and axial direction and φ is the angle between the fiber reinforcement and the circumferential direction in individual layers; $c_{1}$ , $c_{2}$ , $D_{1}$ , $D_{2}$ , $K_{1}$ and $K_{2}$ are material parameters. Parameter values for the vessel layers and plaque components used in our models are: Intima: $c_{1}$ = −169.23 kPa, $c_{2}$ = 177.40 kPa, $D_{1}$ = 2.4 kPa, $D_{2}$ = 13, $K_{1}$ = 32 kPa, $K_{2}$ = 36; Media: $c_{1}$ = −67.25 kPa, $c_{2}$ = 35.01 kPa, $D_{1}$ = 17 kPa, $D_{2}$ = 2, $K_{1}$ = 7 kPa, $K_{2}$ = 4, *φ*= 24.9°; Adventitia: $c_{1}$ = −94.44 kPa, $c_{2}$ = 102.42 kPa, $D_{1}$ = 0.8 kPa, $D_{2}$ = 10, $K_{1}$ = 10 kPa, $K_{2}$ = 40, *φ*= 75.3°; lipid core: $c_{1}$ = 0.5 kPa, $c_{2}$ = 0 kPa, $D_{1}$ = 0.5 kPa, $D_{2}$ = 1.5; calcification: $c_{1}$ = 920 kPa, $c_{2}$ = 0 kPa, $D_{1}$ = 360 kPa, and $D_{2}$ = 2.0. Stress–stretch curves of three layers used in finite element modelling are given in Figure 2, and are consistent with that in the current literature (Holzapfel et al., 2005; Lv et al., 2021; Wang, L et al., 2021).

### 2.3 Multilayer 3D thin-slice model

Three-dimensional (3D) thin-slice models were constructed for 200 OCT slices from 20 patient using automatically segmented slices obtained from our programs. Both multilayer and single-layer models were constructed for each slice, resulting in 400 thin-slice models in total. The 3D thin-slice model was made by adding a 0.5 mm thickness to each slice so that the 3D thin-slice solutions would be better approximations to full 3D models, yet the thin-slice model construction cost is about the same as that of 2D models. Since OCT data were acquired under *in vivo* conditions when the vessel was axially stretched and under *in vivo* pressure, a 5% axial shrink–stretch process and a circumferential pre-shrink process were performed to obtain initial stress/strain conditions when *in vivo* slice morphology was recovered under pressure and axial stretch (Wang, L et al., 2021). Finite element mesh was generated using a commercial finite-element package ADINA 9.6 (Adina R and D, Watertown, MA, United States). Since plaques have complex morphologies, a “volume-fitting” technique was introduced to divide the 3-D plaque, intima, media and adventitia domains into hundreds of small “volumes” to curve-fit the irregular vessel geometry with plaque component inclusions (Yang et al., 2009). This technique was essential in getting convergent plaque finite element models. Mesh analysis was performed by decreasing mesh size by 10% (in each dimension) until solution differences were less than 2%. The mesh was then chosen for our simulations. The thin-slice models were solved following our established procedures (Lv et al., 2021). Because stress/strain are tensors, maximum principal stress and maximum principal strain (called stress and strain from here on, respectively) were chosen as their scale representatives for stress/strain comparisons.

### 2.4 Data extraction and analysis

**FIGURE 3:**
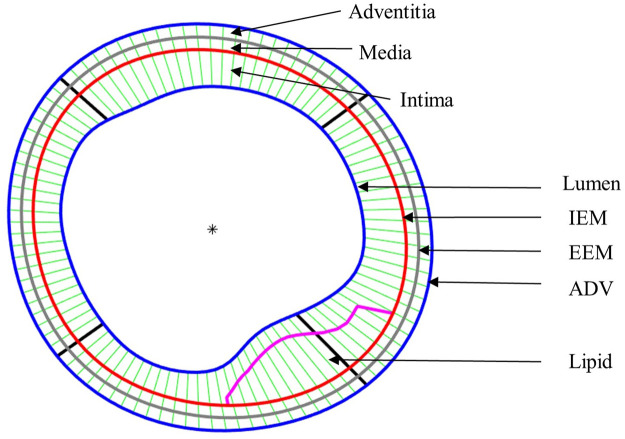
Schematic plot demonstrating the Piecewise Equal-Step method for three layers and the quarter-dividing method. IEM, Internal elastic membrane; EEM, External elastic membrane; ADV, adventitia-periadventitia interface.

After plaque models were solved, stress and strain results at plaque inner wall (called plaque stress/strain for simplicity), cap (cap stress/strain) and out-wall (out-wall stress/strain) were extracted to compare multilayer and single-layer model results and investigate the impact of three-layer segmentation on plaque stress/strain calculations. Since plaque slices often have irregular and nonuniform wall thickness, a four-quarter method (see Figure 3) was introduced to connect lumen points and out-wall points to avoid data distortion by thicker plaques (Wang, Q et al., 2019). Figure 3 gives an illustration of the four-quarter method and the three layers of the vessel: intima, media and adventitia. The boundary between intima and media is called internal elastic membrane (IEM), which is a thin membrane mainly composed of elastin. The boundary between media and adventitia is called external elastic membrane (EEM). The boundary between adventitia and other peripheral tissues is called adventia-periadventitia interface (ADV).

It is commonly agreed that high stress/strain conditions may be associated with plaque ruptures. Therefore, maximum plaque stress/strain values from multi- and single-layer models were compared. It is also of interest to compare mean stress/strain values to see model differences. Ten slices were chosen for each plaque to construct models and each slice have 100 nodal points. That brought a total of 1,000 nodal points for each plaque. Equations 3, 4 were used to obtain maximum and mean plaque stress of each patient, respectively. Calculations for maximum and mean plaque strain were similar. Eq. 5 was used to calculate the relative difference of patient maximum plaque stress between multi- and single-layer models. Relative differences of other quantities were calculated similarly. Equations 6, 7 were used to calculate average maximum plaque stress (strain) and average mean plaque stress (strain) for all 20 patients.

$$Patient\;Maximum\;Plaque\;Stress=\max_{i=1,2\cdots 1000}\left\{plaque\;stress\;\left(i\right)\right.\}$$ (3)

$$Patient\;mean\;plaque\;stress=\frac{1}{1000}\sum_{i=1}^{1000}plaque\;stress\;\left(i\right)$$ (4)

Patient maximum stress difference=

$$\frac{Patient\;maximum\;plaque\;stress\left(Multi\right)-Patient\;maximum\;plaque\;stress\left(Single\right)}{Patient\;maximum\;plaque\;stress\left(Single\right)}\times 100\%$$ (5)

$$Average\;Maximum\;Plaque\;Stress=\frac{1}{20}\sum_{j=1}^{20}Patientmaximumplaquestress\;\left(j\right)$$ (6)

$$Average\;mean\;plaque\;stress=\frac{1}{20}\sum_{j=1}^{20}Patient\;mean\;plaque\;stress\;\left(j\right)$$ (7)

The stress and strain data of nodal points in various regions were also extracted and analyzed to examine model differences at different locations. The computation of maximum and mean cap stress considered all cap nodes, while the computation of maximum and mean out-wall stress incorporated all nodes on the out-wall. The formulas calculating cap stress and wall stress were determined in a similar fashion to plaque stress, but have been omitted here for simplicity.

### 2.5 Statistical analysis

The maximum and mean stress/strain of plaque, cap and vessel out-wall location were compared to obtain the differences between multilayer and single-layer thin-slice models. Single-layer data were used as the base when computing relative differences. The Kolmogorov–Smirnov tests were performed to check data normality. Analysis of variance and paired t tests were used to check if the differences between multilayer and single-layer data sets were statistically significant. A *p*-value <0.05 was considered statistically significant.

## 3 Results

### 3.1 Maximum and mean plaque stresses from multilayer models were 42.2% and 38.9% higher than those from single-layer models

Table 2 compared maximum and mean plaque stress values from multilayer and single-layer models from 20 patients. Maximum and mean stress values and difference from the two models for each patient were calculated using formulas presented in the method section. Patient-averaged maximum and mean stress values and model differences are given at the end of the table, accompanied by their standard deviations. The average maximum plaque stress values of 20 patients from multilayer and single-layer models were 385.13 ± 110.09 kPa and 270.91 ± 95.86 kPa, respectively. The relative difference was 42.16%, with single-layer stress serving as the base value (*p* < 0.001). The average mean plaque stress values from multilayer and single-layer models were 129.59 ± 32.77 kPa and 93.27 ± 18.20 kPa, respectively. The relative difference between the two models was 38.93%, slightly less than that for the average maximum plaque stresses (*p* < 0.001). Both maximum and mean plaque stress values and model differences exhibited substantial variations among individual patients. Specifically, maximum plaque stress differences ranged from 14.81% to 89.26%, while mean plaque stress differences spanned from 13.79% to 60.87%. These findings suggest that use of multilayer model could improve plaque stress calculation accuracy and may have large impact on various stress-related plaque research and clinical applications (plaque progression, rupture and patient management).

**TABLE 2:** Maximum and mean plaque stress comparisons between multilayer and single-layer models based on results from 20 patients.

Patient	Maximum plaque stress			Mean plaque stress		
	Multilayer (kPa)	Single-layer (kPa)	Difference (%)	Multilayer (kPa)	Single-layer (kPa)	Difference (%)
1	467.36	279.88	66.99	176.91	109.97	60.87
2	381.40	277.28	37.55	146.13	103.47	41.24
3	289.47	201.25	43.84	118.20	86.70	36.34
4	261.41	173.43	50.73	101.99	77.71	31.25
5	362.32	240.66	50.55	160.32	100.50	59.51
6	388.89	323.54	20.20	150.27	114.24	31.53
7	684.90	523.80	30.76	190.35	139.14	36.80
8	490.42	319.88	53.31	165.07	104.52	57.92
9	322.70	199.48	61.77	96.91	71.58	35.39
10	504.88	316.96	59.29	166.36	106.23	56.59
11	198.05	118.12	67.67	95.64	73.59	29.96
12	408.69	290.87	40.51	117.94	89.84	31.28
13	340.09	251.97	34.97	131.55	96.28	36.63
14	305.10	161.21	89.26	139.06	95.83	45.11
15	432.01	316.37	36.55	150.62	110.79	35.96
16	321.55	257.06	25.09	120.71	88.56	36.30
17	262.60	140.37	87.07	104.65	74.08	41.27
18	486.95	424.14	14.81	87.11	74.64	16.71
19	355.37	268.27	32.47	78.06	68.60	13.79
20	438.45	333.73	31.38	93.89	79.17	18.59
Mean ± SD	385.13 ± 110.09	270.91 ± 95.86	42.16	129.59 ± 32.77	93.27 ± 18.20	38.93

### 3.2 Maximum and mean plaque strains from multilayer models were 11.6% and 19.0% higher than those from single-layer models

Table 3 compared maximum and mean plaque strain values from multilayer and single-layer models from 20 patients. Strain differences between the two models were smaller than stress differences. The average maximum plaque strain values were 0.264 ± 0.066 and 0.237 ± 0.069 for multilayer and single-layer models, with a relative difference of 11.57% (*p* < 0.001). The average mean plaque strain values were 0.133 ± 0.019 and 0.112 ± 0.015 for multilayer and single-layer models, with a relative difference of 19.02% (*p* < 0.001).

**TABLE 3:** Maximum and mean plaque strain comparisons between multilayer and single-layer models based on results from 20 patients.

Patient	Maximum plaque strain			Mean plaque strain		
	Multilayer	Single-layer	Difference (%)	Multilayer	Single-layer	Difference (%)
1	0.247	0.223	10.90	0.158	0.126	25.44
2	0.285	0.253	12.68	0.146	0.124	17.90
3	0.294	0.255	15.29	0.134	0.112	19.24
4	0.202	0.174	15.62	0.125	0.105	18.54
5	0.219	0.191	14.92	0.150	0.117	27.99
6	0.271	0.259	4.82	0.149	0.130	14.46
7	0.342	0.319	7.25	0.166	0.145	14.52
8	0.227	0.199	13.93	0.151	0.119	26.86
9	0.241	0.205	17.68	0.113	0.092	22.15
10	0.275	0.224	22.81	0.149	0.121	23.33
11	0.174	0.141	23.69	0.115	0.094	21.94
12	0.299	0.270	10.80	0.126	0.108	16.73
13	0.231	0.205	12.62	0.138	0.118	16.12
14	0.198	0.171	15.85	0.142	0.116	22.84
15	0.253	0.231	9.63	0.145	0.127	13.89
16	0.272	0.253	7.55	0.121	0.100	20.82
17	0.192	0.163	17.99	0.120	0.095	26.66
18	0.473	0.461	2.66	0.109	0.099	10.74
19	0.307	0.287	7.12	0.101	0.093	8.15
20	0.286	0.258	10.82	0.108	0.097	11.37
Mean ± SD	0.264 ± 0.066	0.237 ± 0.069	11.57	0.133 ± 0.019	0.112 ± 0.015	19.02

### 3.3 Maximum and mean cap stresses from multilayer models were 36.4% and 28.7% higher than those from single-layer models

Since plaque rupture often happens at thin plaque cap, special attention was given to cap stress and strain conditions. Table 4 compares the maximum and mean cap stress values from multilayer and single-layer models from 20 patients. The average maximum and mean cap stress values for multilayer models were 340.42 ± 148.92 kPa and 119.86 ± 45.46 kPa. The average maximum and mean cap stress values for single-layer models were 249.61 ± 108.09 kPa and 93.16 ± 26.74 kPa. The relative differences for the average maximum and mean stress between two models were 36.38% and 28.66%, respectively (*p* < 0.001). This was slightly smaller than plaque stress differences (42.16% and 38.93%). Additionally, it is important to note that the location of maximum plaque stress may occur either at cap or within normal vessel tissues. Consequently, maximum cap stress could be equal to or smaller than the maximum plaque stress from the entire plaque segment modeled.

**TABLE 4:** Maximum and mean cap stress comparisons between multilayer and single-layer models based on results from 20 patients.

Patient	Maximum cap stress			Mean cap stress		
	Multilayer (kPa)	Single-layer (kPa)	Difference (%)	Multilayer (kPa)	Single-layer (kPa)	Difference (%)
1	467.36	279.88	66.99	212.51	131.23	61.93
2	381.40	277.28	37.55	103.76	82.69	25.47
3	289.47	201.25	43.84	131.38	96.25	36.49
4	164.02	129.00	27.15	86.71	71.15	21.87
5	362.32	240.66	50.55	147.76	102.58	44.05
6	388.89	323.54	20.20	149.10	127.78	16.68
7	684.90	523.80	30.76	228.43	168.23	35.79
8	490.42	319.88	53.31	186.65	122.68	52.15
9	322.70	199.48	61.77	91.47	73.05	25.20
10	504.88	316.96	59.29	133.75	92.06	45.28
11	100.67	92.55	8.77	69.77	62.73	11.23
12	408.69	290.87	40.51	98.57	82.11	20.04
13	158.97	135.50	17.32	80.57	71.19	13.17
14	205.56	128.05	60.53	107.36	82.71	29.80
15	289.30	227.39	27.23	86.39	75.52	14.39
16	197.51	169.89	16.26	84.78	79.34	6.86
17	169.42	139.93	21.08	72.49	63.84	13.55
18	486.95	424.14	14.81	92.27	80.86	14.11
19	296.57	238.49	24.35	105.04	90.65	15.87
20	438.45	333.73	31.38	128.35	106.53	20.48
Mean ± SD	340.42 ± 148.92	249.61 ± 108.09	36.38	119.86 ± 45.46	93.16 ± 26.74	28.66

### 3.4 Maximum and mean cap strains from multilayer models were 9.6% and 12.9% higher than those from single-layer models

Table 5 compared maximum and mean cap strain values from multilayer and single-layer models from 20 patients. The average maximum cap strain values were 0.246 ± 0.077 and 0.225 ± 0.074 for multilayer and single-layer models, with a relative difference of 9.63% (*p* < 0.001). The average mean cap strain values were 0.118 ± 0.028 and 0.105 ± 0.022 for multilayer and single-layer models, with a relative difference of 12.93% (*p* < 0.001).

**TABLE 5:** Maximum and mean cap strain comparisons between multilayer and single-layer models based on results from 20 patients.

Patient	Maximum cap strain			Mean cap strain		
	Multilayer	Single-layer	Difference	Multilayer	Single-layer	Difference
1	0.247	0.223	10.90%	0.168	0.137	22.12%
2	0.285	0.253	12.68%	0.091	0.084	7.91%
3	0.294	0.255	15.29%	0.142	0.121	17.59%
4	0.175	0.161	8.94%	0.109	0.095	14.78%
5	0.213	0.185	15.25%	0.135	0.114	18.84%
6	0.271	0.259	4.82%	0.146	0.136	7.46%
7	0.342	0.319	7.25%	0.178	0.158	12.58%
8	0.227	0.199	13.93%	0.151	0.125	20.82%
9	0.241	0.205	17.68%	0.104	0.090	15.23%
10	0.245	0.215	14.32%	0.114	0.096	19.03%
11	0.133	0.125	6.69%	0.086	0.078	9.85%
12	0.299	0.270	10.80%	0.103	0.094	8.84%
13	0.180	0.163	9.84%	0.104	0.096	8.43%
14	0.175	0.152	14.87%	0.114	0.096	18.05%
15	0.200	0.185	8.10%	0.098	0.091	7.59%
16	0.176	0.182	−3.71%	0.074	0.073	0.91%
17	0.177	0.163	8.49%	0.093	0.083	12.96%
18	0.473	0.461	2.66%	0.112	0.104	7.78%
19	0.285	0.259	10.09%	0.116	0.107	8.15%
20	0.286	0.258	10.82%	0.133	0.120	10.75%
Mean ± SD	0.246 ± 0.077	0.225 ± 0.074	9.63%	0.118 ± 0.028	0.105 ± 0.022	12.93%

### 3.5 Maximum and mean plaque out-wall stresses from multilayer models were 55.1% and 71.6% lower than those from single-layer models

**FIGURE 4:**
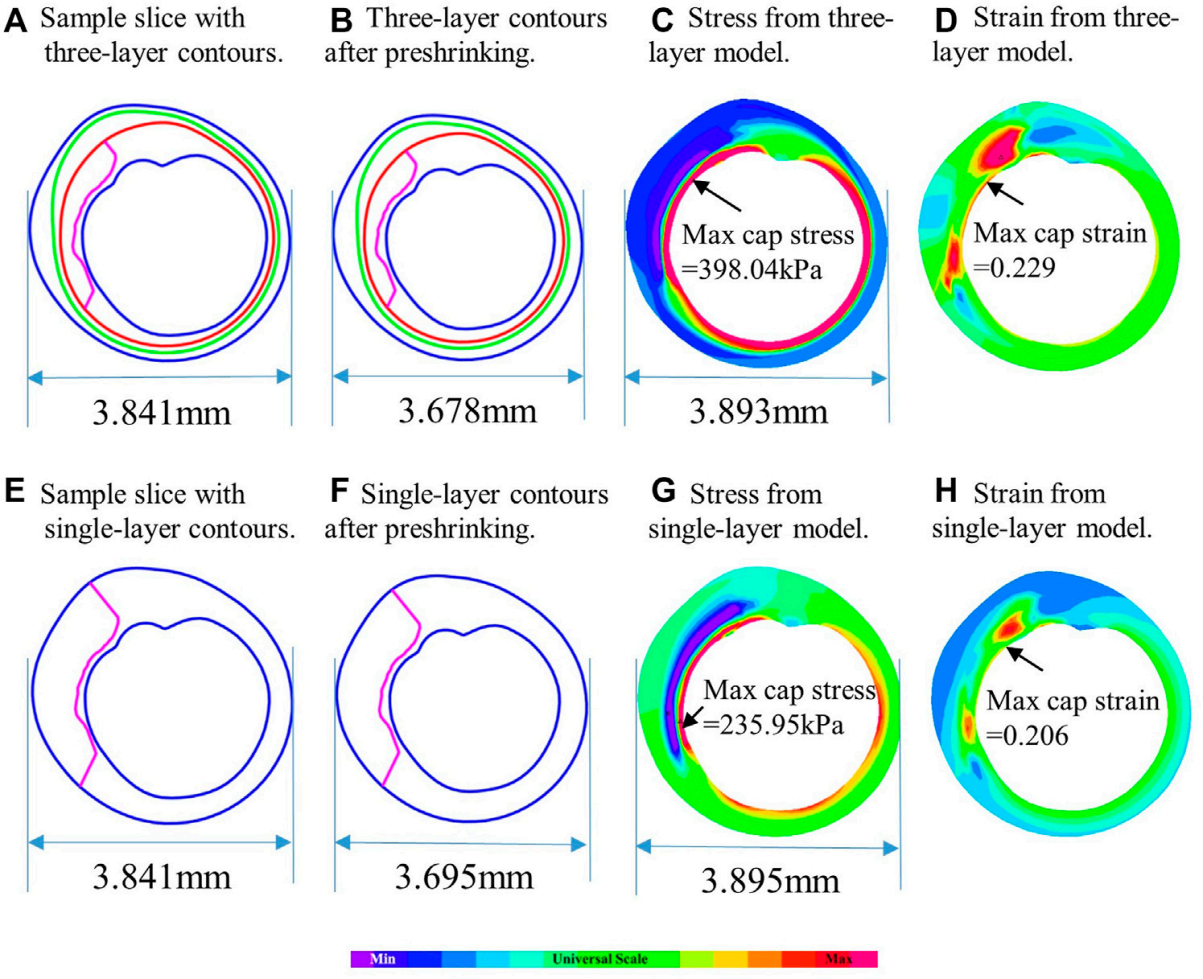
Comparison of plaque stress/strain distributions from multilayer and single-layer models using a sample slice. **(A)** Sample slice with multilayer contours. **(B)** Multilayer contours after preshrinking. **(C)** Stress from multilayer model. **(D)** Strain from multilayer model. **(E)** Sample slice with single-layer contours. **(F)** Single-layer contours after preshrinking. **(G)** Stress from single-layer model. **(H)** Strain from single-layer model.

Table 6 showed that average maximum out-wall stress as 34.76 ± 148.92 kPa for multilayer models and 77.38 ± 12.00 kPa for single-layer models, with a relative difference of −55.07% (*p* < 0.001). The average mean out-wall stress values were 14.62 ± 4.54 kPa and 51.74 ± 7.73 kPa for multi- and single-layer models, yielding a relative difference of −71.58% (*p* < 0.001). Additionally, the sign shifted from positive to negative, indicating that single-layer models considerably over-estimated out-wall stresses. Multilayer models had greater inner-wall plaque stresses and reduced out-wall stresses. Figure 4 also demonstrated this stress distribution difference between multilayer and single-layer models.

**TABLE 6:** Maximum and mean wall stress comparisons between multilayer and single-layer models based on results from 20 patients.

Patient	Maximum out-wall stress			Mean out-wall stress		
	Multilayer (kPa)	Single-layer (kPa)	Difference	Multilayer (kPa)	Single-layer (kPa)	Difference
1	33.63	79.42	−57.66%	18.77	58.84	−68.11%
2	63.73	89.32	−28.65%	18.35	58.49	−68.63%
3	31.43	80.73	−61.07%	13.82	50.71	−72.74%
4	24.03	64.34	−62.65%	11.05	45.75	−75.85%
5	30.92	77.76	−60.23%	21.42	62.24	−65.58%
6	32.45	80.03	−59.45%	12.83	49.07	−73.85%
7	28.92	72.13	−59.90%	15.61	55.05	−71.65%
8	54.21	91.84	−40.98%	20.54	59.48	−65.46%
9	34.88	76.54	−54.44%	10.54	43.88	−75.97%
10	43.74	86.38	−49.37%	21.83	63.08	−65.39%
11	27.53	71.38	−61.43%	13.24	48.69	−72.80%
12	35.35	89.72	−60.60%	12.96	49.75	−73.96%
13	32.38	81.33	−60.19%	14.84	52.62	−71.80%
14	42.95	93.20	−53.91%	20.20	60.78	−66.77%
15	39.77	91.31	−56.45%	17.19	57.23	−69.97%
16	27.49	68.31	−59.76%	12.26	46.76	−73.77%
17	39.40	81.64	−51.74%	13.74	48.63	−71.74%
18	24.90	62.47	−60.14%	8.61	40.85	−78.92%
19	14.85	47.79	−68.94%	6.34	36.80	−82.78%
20	32.73	62.00	−47.21%	8.34	40.61	−79.48%
Mean ± SD	34.76 ± 10.77	77.38 ± 12.00	−55.07%	14.62 ± 4.54	51.47 ± 7.73	−71.58%

### 3.6 Maximum and mean out-wall strains from multilayer models were 106.4% and 47.0% higher than those from single-layer models

Table 7 compared the maximum and mean out-wall strain values from multilayer and single-layer models from 20 patients. The average maximum out-wall strain values were 0.161 ± 0.030 and 0.078 ± 0.015 for multilayer and single-layer models, with a relative difference of 106.40% (*p* < 0.001). The average mean out-wall strain values were 0.079 ± 0.018 and 0.054 ± 0.002 for multilayer and single-layer models, with a relative difference of 46.95% (*p* < 0.001). The relative differences were substantially larger than that observed for inner-layer plaque and cap strains.

**TABLE 7:** Maximum and mean out-wall strain comparisons between multilayer and single-layer models based on results from 20 patients.

Patient	Maximum out-wall strain			Mean out-wall strain		
	Multilayer	Single-layer	Difference (%)	Multilayer	Single-layer	Difference (%)
1	0.171	0.078	118.02	0.099	0.055	81.22
2	0.212	0.096	121.27	0.092	0.055	67.04
3	0.158	0.080	96.23	0.072	0.053	36.12
4	0.131	0.061	115.68	0.060	0.051	17.56
5	0.173	0.072	139.94	0.117	0.054	114.91
6	0.151	0.073	107.86	0.072	0.052	36.37
7	0.166	0.072	130.64	0.089	0.052	72.57
8	0.211	0.098	115.70	0.101	0.057	79.30
9	0.161	0.081	98.70	0.064	0.052	22.80
10	0.201	0.098	103.95	0.106	0.056	88.76
11	0.140	0.069	104.20	0.072	0.053	36.09
12	0.160	0.090	78.01	0.073	0.054	35.58
13	0.159	0.080	98.89	0.078	0.055	41.90
14	0.175	0.104	68.78	0.096	0.059	63.32
15	0.156	0.094	65.19	0.082	0.057	42.42
16	0.140	0.065	115.72	0.065	0.052	27.00
17	0.155	0.082	88.18	0.072	0.053	35.93
18	0.106	0.061	71.95	0.055	0.051	7.11
19	0.101	0.051	97.30	0.054	0.051	4.54
20	0.190	0.053	261.90	0.058	0.051	13.68
Mean ± SD	0.161 ± 0.030	0.078 ± 0.015	106.40	0.079 ± 0.018	0.054 ± 0.002	46.95

## 4 Discussion

### 4.1 Significance of multilayer coronary plaque models and layer-specific material properties

While it is generally believed that plaque cap stress/strain may be closely related to plaque rupture and vulnerability, it is yet to be validated by large-scale studies to establish direct linkage between plaque stress/strain conditions and several clinical cardiovascular events. The three-layer model introduced in this paper improved the accuracy of stress/strain calculations. Whether maximum and mean stress/strain could be chosen as the main parameters for vulnerability assessment still need to be validated by large-scale studies.

In recent decades, there has been a concerted focus on developing patient-specific coronary plaque models based on imaging data to analyze the mechanical conditions of plaques and investigate the impact of plaque morphological and mechanical conditions on plaque progression and potential rupture. Most plaque models in the literature are one-layer models. However, it is important to emphasize the critical role of using accurate vessel layered structure and material properties in obtaining reliable plaque mechanical conditions. In previous models, the three-layer vessel structure and layer-specific vessel materials were often overlooked because of limited imaging resolution. With the development in medical imaging technology and advancements in imaging modalities, OCT has revolutionized imaging resolution and enables the differentiation of the three-layer vessel structure based on image intensity variations for the first time (Ali et al., 2016). This breakthrough has opened up new possibilities for developing patient-specific multilayer coronary plaque models and incorporating layer-specific vessel material properties. This paper used segmented three-layer OCT coronary plaque data and constructed multilayer models to improve the accuracy of plaque stress and strain calculations. This advancement in modeling has great potential for improving predictions of plaque progression and vulnerability.

### 4.2 Multilayer and single-layer models demonstrated large stress/strain differences

Results from our paper demonstrated that plaque stress and strain values from multilayer and single layer coronary plaque models had large significant differences. The maximum and mean plaque stresses from the multilayer models were 42.2% and 38.9% higher than those from the single-layer models. Similarly, the maximum and mean cap stresses increased for 36.4% and 28.7% in the multilayer models. This suggests that single-layer models tend to underestimate plaque and cap stress, potentially leading to an underestimation of future risk of plaque rupture.

Regarding plaque strain values, the maximum and mean plaque strains obtained from the multilayer models were 11.6% and 19.0% higher than those from single layer models. Similarly, the maximum and mean cap strains showed increases of 9.6% and 12.9%. It should be noted that differences in model strain values were smaller than those for stress value differences.

Interestingly, the investigation of out-wall stress and strain yielded contrasting results. The maximum and mean out-wall stresses in the multilayer models were significantly lower, with reductions of 55.1% and 71.6%, respectively. However, the maximum and mean cap strains showed increases of 106.4% and 47.0% in the multilayer models. This proves that single-layer models overestimate out-wall stress and underestimate out-wall strain. This was caused by differences in layer material properties.

Validations of computational models are generally done by *in vitro* experiments where three-layer vessels with specific material properties are made and are subjected to specified stretch pressure conditions to obtain vessel displacements. Results from computational models are compared with the experimental results to seek model validation. Our modeling approach and ADINA models were validated by *in vitro* experiments at the beginning of our long-term effort investigating coronary biomechanics (Ji et al., 2008; Yang et al., 2008). Validation of our three-layer models by *in vitro* experiments is our future effort with available resources.

Validation of simulation results, i.e., differences in stress/strain results from three- and single-layer models is built in our modeling process: our modeling approach required that our pressurized vessels matched *in vivo* OCT data as much as possible. This is one way to validate our results. Since both three- and single-layer models were subjected to the same pre-stretch and pressure conditions, our results showing model differences should be considered reliable.

Other factors such as pressure conditions and blood conditions (sugar, cholesterol, viscosity, *etc.*) may also affect model results. Our results about model differences should be interpreted with caution.

### 4.3 Limitations and future directions

Some limitations of this study include: (a) This is a pilot study and the patient data size is small. Further validation through larger-scale studies is necessary to verify our findings. (b) 3D thin-slice models were used in this study to save model construction time. Full 3D models with fluid-structure interaction (FSI) models could be used to improve accuracy of stress/strain calculations. (c) Due to the unavailability of patient-specific vessel material properties, material parameter values from the literature were utilized in this study. There have been studies for obtaining *in vivo* vessel material properties using IVUS or OCT (Wang, L et al., 2021; Guo, Q et al., 2023). In the future, our model could include patient-specific vessel material properties with the aid of advanced imaging modalities and techniques. (d) Residual stress was not considered in this study. Previous studies showed that residual stress is also layer-specific, and each layer has different opening angle (Holzapfel et al., 2007). Future study may take layer-specific residual stress into consideration to obtain more accurate model results.

## Data Availability

The original contributions presented in the study are included in the article/Supplementary Material, further inquiries can be directed to the corresponding authors.

## Abbreviations

ADV, adventitia-periadventitia interface; BP, blood pressure; DM, diabetes mellitus; EEM, External elastic membrane; HL, hyperlipoproteinemia; HT, hypertension; IEM, Internal elastic membrane; IVUS, Intravascular ultrasound; LAD, left anterior descending artery; LCX, left circumflex artery; OCT, Optical coherence tomography; RCA, right coronary artery; 3D, Three dimensional.
